# Interferon-Based Therapy for Chronic Hepatitis C: Current and Future Perspectives

**Published:** 2010-09-01

**Authors:** Valli De Re

**Affiliations:** 1 Experimental and Clinical Pharmacology, FSC, DOMERT, Centro di Riferimento Oncologico, IRCCS, National Cancer Institute, Aviano, Italy

Dear Editor,

We greatly enjoyed reading the excellent meta-analysis by Alavian and colleagues [1]on the efficacy and safety of peginterferon (PEG-IFN) alpha-1a vs. 2b for the treatment of chronic hepatitis C virus (HCV) infection.The immune effects of interferons, often in combination with other drugs, have been utilized to treat several diseases, such as HCV and HCV-related autoimmune diseases and liver diseases. Several different types of interferon are approved for use in humans. For instance, in January 2001 the Food and Drug Administration approved the use of pegylated interferon-alpha; in this formulation, polyethylene glycol is added to make the interferon last longer in the body. Peginterferon alpha-2b (Pegintron) was the first treatment approved for public use, and approval for peginterferon alpha-2a (Pegasys), which has a longer duration in serum, followed in October 2002. When used with the antiviral drug ribavirin, pegylated interferon is effective in the treatment of HCV; however, up to now, there is no convincing clinical evidence that peginterferon alpha-2a has a different response than peginterferon alpha-2b treatment. In the meta-analysis reported herein,  peginterferon alpha-2a showed an early virological response at treatment week 4 (EVR) and a sustained virological response (SVR) at week 12, and alpha-2a had a better response than did alpha-2b in patients with HCV genotypes 1-4 (OR 1.38) and genotype 2 (OR 4.06). These data are of interest because single studies conducted before 2009 suggested a trend towards peginterferon alpha-2a, but the treatment reached significance only when several studies were pooled together (hereafter the meta-analysis refers to 3,518 grouped patients). In addition, the review also offers important information regarding safety, indicating a difference among the two treatments in only the causation of neutropenia (<750c/mm3), with an overall estimate of OR 0.75 of discontinuing the peginterferon alpha-2a treatment. Usually, the use of a granulocyte colony stimulating factor is effective in correcting neutropenia [2][3].Thus, the review provides important information about the clinical steps and best practices in the treatment of chronic HCV infection with peginterferon alpha-2a. A limitation of the study is that no data were reported on liver histology, an area that should be explored in the future.Furthermore, despite significant progress in HCV antiviral therapy, tolerance for and compliance with PEG-IFN/Ribavirin therapy remain a serious problem because, as reported by the authors, the treatment may induce a sustained clearance of the virus and clinical improvement in a median of 75% people with HCV genotypes 2 or 3, but the treatment is effective in less than 50% of people infected with genotype 1, the most common form of HCV in both the United States and Western Europe. Thus, future perspectives will be focused on developing new drugs; indeed, the most in advanced clinical developments are direct antiviral agents. The NS3 protease, essential for viral replication, represents an important target for these therapies. The clinical efficacy of NS3 protease inhibitors was first demonstrated with compounds targeting the enzyme active site BILN 2061, boceprevir (SCH 503034), and telaprevir (VX-950) in monotherapy and in combination with PEG-IFN. During boceprevir or telaprevir monotherapy, multiple resistance mutations were selected [4][5]. Therefore, combination therapy with pegylated interferon-alfa, ribavirin, or other direct antiviral drugs seems mandatory to avoid the development of resistance. The safety and efficacy of plus peginterferon alfa-2b and ribavirin in the treatment of naïve patients with chronic hepatitis C genotype 1 infection showed response rates of up to 75% for boceprevir and 68% for Telaprevir [6][7], but the underlying mechanism of the response to the treatment is still not clear. Thus, although encouraging clinical results have been achieved with these first-generation protease inhibitors, additional improvement in inhibitor potency and pharmacokinetic properties will be pursued to increase SVR. Moreover, predictive biomarkers of poor virological response to the treatments will be investigated in amino acid (aa) substitution studies of host genes [8], analyses of HCV gene regions of infected patients [9][10], and/or the role of drugs on host immune responses such as upregulation of major histocompatibility complex molecules, increases in immunoproteosome activity, and the activation of certain immune cells (e.g., macrophages and natural killer cells). In addition, the evidence up to this point has indicated that HCV can bind and internalize into primary B-cells; furthermore, one analysis found that HCV failed to create a productive infection in these cells, and the virus showed in vitro an enhanced infectivity compared with the extracellular virus and an ability to promote B-cell adhesion to hepatoma cells [11]. Thus, the role of B-cell elimination, which serves as long-term HCV reservoirs, should be considered in the future. With respect to this topic, one of our recent papers [12] noted a significant depletion of inflammatory cells and a tricking regression of hepatocytolytic foci in histological liver samples obtained at the end of a PEG-IFN/Ribavirin, plus rituximab treatment (PIRR treatment). The results of the study thus indicated that PIRR, including B-cell depletion due to the addition of a monoclonal anti-CD20 antibody, rituximab, in anti-HCV therapy can establish a prolonged histological remission rather than an increased rate of eradication of HCV infection as compared with PEG-IFN/Ribavirin combination, at least in patients with cryoglobulinemia.
